# An insider’s guide to understanding and obtaining an NIH K career development award

**DOI:** 10.1172/jci.insight.191904

**Published:** 2025-06-23

**Authors:** Don C. Rockey, Kyu Y. Rhee, Christopher S. Williams, Jatin M. Vyas, Charles W. Emala, Emily J. Gallagher

**Affiliations:** 1 Digestive Disease Research Center, Medical University of South Carolina, Charleston, South Carolina, USA.; 2 Division of Infectious Diseases, Department of Medicine, Weill Cornell Medicine, New York, New York, USA.; 3 Division of Gastroenterology, Department of Medicine, Vanderbilt University Medical Center, Nashville, Tennessee, USA.; 4 Veterans Affairs Tennessee Valley Health Care System, Nashville, Tennessee, USA.; 5 Division of Infectious Disease, Department of Medicine, and; 6 Department of Anesthesiology, Vagelos College of Physicians and Surgeons, Columbia University, New York, New York, USA.; 7 Division of Endocrinology, Diabetes and Bone Disease, Department of Medicine, Icahn School of Medicine at Mount Sinai, New York, New York, USA.

## Abstract

Physician-scientists in academic medical centers require extramural grant support to launch and maintain their research careers. In order to cultivate the next generation of biomedical researchers, including physician-scientists, the NIH supports multiple career development (K series) awards. For many, their first experience in grant writing is composing a career development award (CDA) application. From the applicant’s perspective, this process can be difficult. For one, NIH institute–specific differences between the same K mechanism can be confusing. Additionally, the importance of the various elements that make up the K application are frequently misunderstood. Furthermore, many K applications will not be funded on the initial submission; therefore, the need to resubmit an application should not be viewed as a sign of failure, but rather can be viewed as an element of resilience in biomedical research. In this piece, we aim to provide guidance for aspiring K applicants — in particular, from the reviewer perspective — with the intent of making the application process more understandable. We offer dos and don’ts on different components of the K application, advice on when to reach out to a program officer, and tips on resubmission. Our overarching goal is to provide support for prospective K applicants in their effort to obtain a K award. While targeted to K applications, most of the contents of this summary apply to any CDA.

## Abstract 2

This manuscript provides advice to physician-scientist Applicants wishing to apply for an NIH K award, with a goal to provide support for prospective K Applicants.

## Graphical abstract

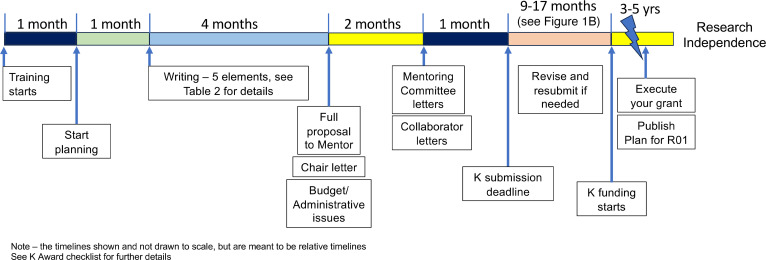

## Introduction

Grant writing is an essential skill for researchers of all career stages, including those seeking funding for specific projects and also those seeking to obtain career development support. This manuscript aims to provide a reviewer’s perspective on the best practices for developing a successful NIH K series career development award (CDA) application. We highlight key criteria by which proposals are judged, what reviewers expect, and tips for creating a compelling application. This document was developed as the result of all of the authors’ experiences, including, in many cases, sitting on K-focused study sections. Additionally, insights were gathered at the annual American Society for Clinical Investigation/Alliance for Academic Internal Medicine/Burroughs Wellcome Fund (ASCI/AAIM/BWF) Physician-Scientist Pathways Workshop (1). While the focus of this piece is on K series grants (i.e., NIH grants) with an emphasis on physician-scientists (K08 and K23, and some K99), many of the principal points highlighted herein apply to other types of CDAs, including those from non-NIH funding agencies, as well as international physician-scientist CDA programs.

While the NIH publishes instructions for completing a K series application, it does not provide insights on strategy and best practices for maximizing chances for a successful application. Those writing K series grants, their mentors, and anyone interested in career development should find the content of this helpful. Finally, it should be appreciated that the NIH recently implemented changes intended to simplify, streamline, and improve peer review. These changes will clearly affect R series and F series applications. As of this writing, K series applications have not yet been modified.

There are many reasons for the NIH and other funders to support career development. In the case of physician-scientists, the data warn that this group is threatened in academic medicine (2–9). Therefore, the more support that can be obtained, the better the chances of improving and retaining this vital component of the biomedical workforce. The NIH in particular has placed a substantial focus on career development — and thus committed to funding K awards, including K08/K23 awards, which are targeted to physician-scientists. To maximize the chances of funding success, the applicant must provide clear, compelling, and objective evidence of a commitment to a full-time investigative career, including a mentorship team with clearly defined roles, and a strategically defined and specific career development plan (CDP). It should be recognized that there are often challenges in pursuing a K award, but, in general, the NIH’s goal is to support the development of young scientists. It should also be emphasized that obtaining funding often requires persistence, including revision (one or more) and resubmission.

## Types of NIH grants, including K series grants

There are many types of NIH grants that support career development and/or research; each serve different purposes. The major NIH series include the following (Table 1): Fellowships (F series), Career Development (K series), Training Grants (T series), Research Grants (R series), Program Projects (P series), and Cooperative Agreements (U series) (note that contracts, which can be an additional source of research funding, are not discussed). It is important to emphasize that the scorable criteria for each of these types of NIH awards varies somewhat. It is important to first recognize the distinctions between K series and other NIH funded grants. While sometimes viewed as primarily administrative, these distinctions are often linked to distinct goals and more significantly associated with important, but often overlooked, distinctions in the review criteria used to evaluate each type of grant. Scorable criteria for K Series grants can be found online and include items such as commitment of the applicant to a career in research, the CDP, the mentor (or mentors and, increasingly, the mentorship team), institutional support and commitment to the applicant, and the environment for developing a research career (Table 2). Note that subsequent to January 25, 2025, scorable criteria for R and F type grants changed; as of the current time, the scorable criteria for K awards have not changed. By design, K awards are intended to place emphasis on the potential of the applicant and especially on how the proposal will foster and facilitate the applicant’s career development. Thus, while K award applications must include a strong research plan, those that prioritize the research plan without paying proper attention to the training and career development aspects of the application risk greater scoring emphasis on the potential success of the proposed research, rather than the applicant. The most critical aspect of the research plan is that it should provide an appropriate vehicle for training; however, elements critical to other grant types, such as innovation and specific aspects of the research plan, are typically not reviewed as critically.

The primary K series grants, for which physician-scientists will apply, include the K08, K23, and, in some NIH institutes, K99 (Table 3). In most institutes, the K08 is intended for those engaged in basic science research (“wet bench”), which has some link to clinical medicine. The K23 is intended for those engaged in patient-oriented research (“clinical research”), and usually deals with patient data or patient specimens. It should be noted that there are some differences in Ks across NIH institutes. The K99/R00 is likely the most nuanced application, as it varies from NIH institute to institute. In some institutes, it is highly competitive and is the only K award that does not require the applicant to be a US citizen or permanent resident at the time of the award.

Unfortunately, there is considerable variation among the various K awards in the different NIH institutes. For example, while the K23 award is extremely popular as a mechanism to support physician-scientists focused on clinical and/or translational research for many NIH institutes, the National Cancer Institute (NCI) has phased out K23 awards (in 2018) in favor of institutional K12, K99/R00, and K08 awards. On one hand, although physician-scientists are not prohibited from applying for the K99/R00 mechanism, it is uncommon in many institutes for physician-scientists to be funded via this mechanism. Therefore, applicants interested in a K award should always talk to the appropriate institute program officer about the details of the portfolio of K awards in the institute to which their application will likely be assigned. The K01 award is given primarily to PhD candidates and can be focused on any type of biomedical, behavioral, or clinical science research.

While all K awards receive an impact or priority score, K series grants do not historically have percentile scores that determine funding (though some institutes may provide percentile scores). This, in theory, allows NIH institutes some degree of flexibility when making funding decisions for K awards based on the application’s potential impact. Specific NIH Institutes (e.g., National Heart, Lung, and Blood Institute [NHLBI] and National Institute of Allergy and Infectious Diseases [NIAID]), but not all, publish the historical payline impact scores for K awards. For physician-scientists, who are the primary target of the K08 award, funding rates are high (Table 4, these data do not differentiate between initial applications and initial or repeat resubmission, but the funding rate after resubmission is typically even higher). Although K23 awards fund individuals other than physician-scientists, funding rates for K23 awards are also high (Table 4). Interestingly, an analysis of NIH reporter data found that although K99s made up 31% of K CDAs among K01, K08, K23, and K99 awardees, only 2% of physician-scientist/clinician-investigator K awardees in internal medicine received a K99 (8).

Anyone applying for a K series grant is encouraged to consult with their program officer for information related to their grant application and/or study section comments. Typically, a program officer will request a copy of the Specific Aims page as a conversation starter to best guide the applicant. Questions to ask the program officer range from simple questions, such as who will review the grant, to more detailed questions about the specific science that is proposed or that has been reviewed (Table 5). It is important to emphasize that the vast majority of NIH program officers who oversee K award portfolios are eager to help applicants navigate the K award process; thus, it is strongly encouraged for applicants to interact with them prior to preparing their full proposal.

For K award applicants at institutions with KL2 programs, it is essential to understand whether funding on the KL2 impacts the duration of funding of an individual K award. This again differs by NIH institute, with some institutes (e.g., Eunice Kennedy Shriver National Institute of Child Health and Human Development, National Eye Institute) having 6-year limits to the total duration of KL2 and K08/K23 funding, while for NHLBI, the total is 8 years, but there is no limit at NCI. Therefore, clarifying with the NIH institute’s program officer any limits in duration of the institutional KL2 and individual K awards is important before submitting the K award application.

## Key elements for success

### Timeline considerations for a career development application.

**Figure 1:**
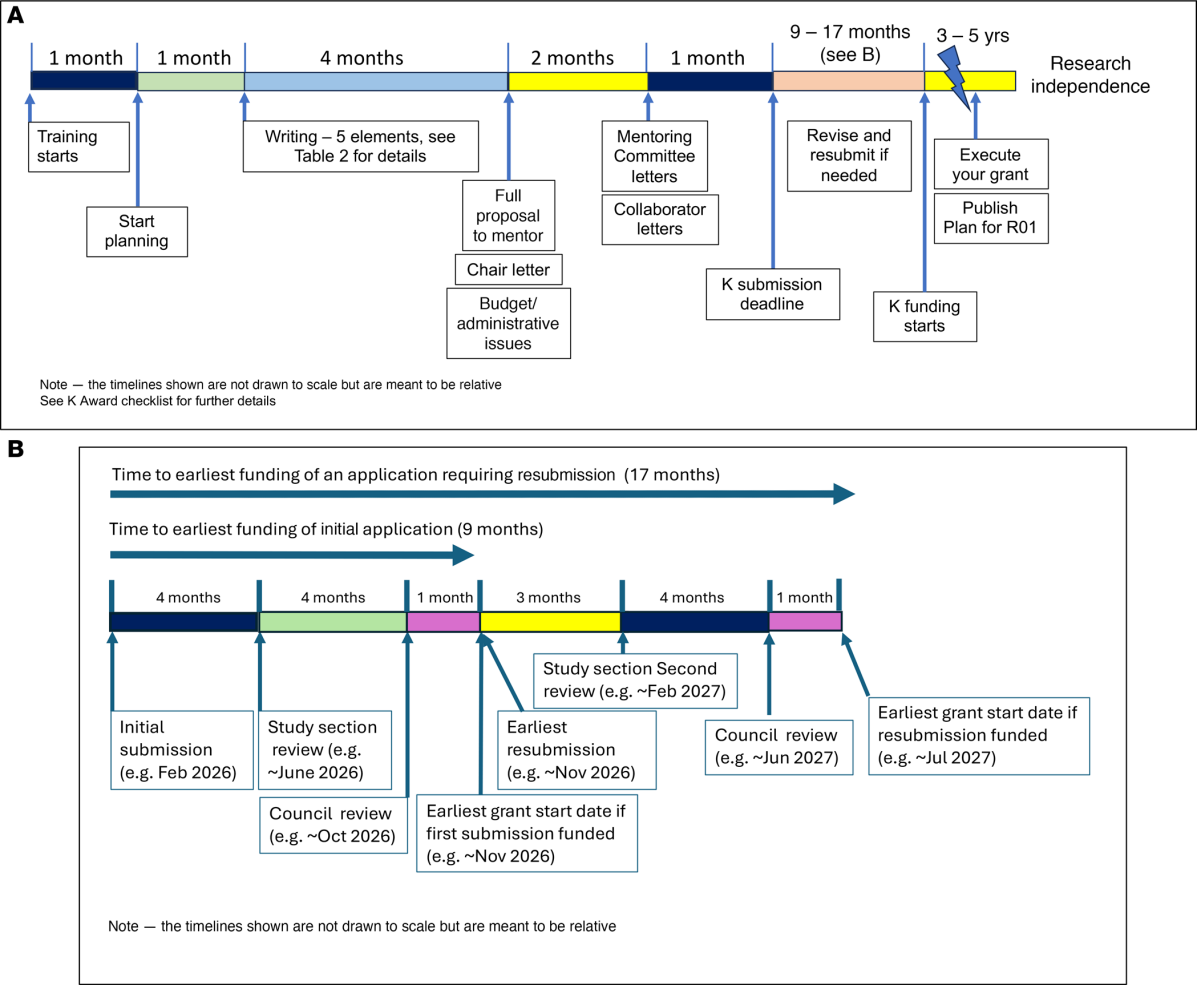
Typical timelines for NIH K award applications. (**A**) The timeline expected for preparation and submission of a K award. (**B**) An expected timeline around a resubmission application.

Careful attention to timing is crucial for a competitive and successful CDA application. Applicants should take into account their career stage and key milestones and closely consult with mentors, institutional leaders, and collaborators when timing the application. While timelines may vary depending on characteristics unique to the applicant, certain elements, including the following, often signal strong readiness: completion or near completion of clinical training, evidence of research productivity, such as a first-author manuscript submitted or accepted, success with internal funding opportunities, society awards, notable accomplishments, and recognition from societies (e.g., the ASCI’s Emerging-Generation Award) as well as clear institutional support, including protected time and committed mentors. In addition to these benchmarks, applicants should plan well in advance — ideally starting 6 months or more before the submission deadline — to allow adequate time for writing, gathering letters, and obtaining internal approvals (Figure 1 and supplemental materials — K Award Checklist; supplemental material available online with this article; https://doi.org/10.1172/jci.insight.191904DS1). Importantly, first-time applicants should be aware that the postsubmission process is lengthy. Even without the need for resubmission, the timeline from submission to a final funding decision often spans 9 months or longer. Understanding this lag is crucial for career planning, especially for those transitioning from fellowship to faculty roles or aligning grant support with the start of protected research time.

### The applicant’s commitment to research.

A strong and consistent demonstration of commitment to research is vital. The application should tell a compelling story about the applicant’s dedication to research, supported by a well-structured CDP and a robust research plan — all of which are well integrated into a cohesive “story.” The personal statement in the biosketch provides an additional chance to emphasize the commitment to a career in research.

### The applicant’s qualifications.

The qualifications of the applicant are an important component of the application. A strong training track record to date always supports an application, and examples not only of outstanding institutional training, but also specific and unique training experiences are helpful; for example, specific molecular biology training in a laboratory during a college, medical school, PhD training, or fellowship provide confidence on the part of the reviewer that molecular biology–type experiments could be readily carried out in a research plan. If in vivo experiments are proposed, previous experience handling animals would enhance the applicant’s qualifications. Evidence of previous individual funding (e.g., F30/F31/F32 or foundation support) or institutional support (T32, R38, R25, KL2, or other awards) during PhD, residency, or fellowship training is often viewed favorably by grant reviewers.

The biosketch is often one of the most overlooked elements of an application. However, a strong candidate [and mentor(s)] biosketch provides an opportunity to tell a compelling story and, perhaps more importantly, highlight specific objective evidence of their commitment to and productivity in science. Key forms of such evidence include peer-reviewed publications, fellowship and honorific awards, national and international presentations, and specific training experiences. The applicant should make it clear to the reviewer that they are committed to research and that they are highly capable of engaging in the proposed career development.

It is additionally important for candidates to be aware that biosketches in K awards can be different than those of their mentors. Furthermore, applicants should be certain to take advantage of these opportunities. For example, in a K award, the applicant may include elements — such as presentations at national meetings — which are typically not allowed in biosketches, but which help emphasize the candidate’s experience, expertise, and qualifications. It is essential that the applicant carefully review NIH guidelines about what is and what is not allowed in a K award application biosketch. Attention to detail is also important in the biosketch (as well as throughout the application). The order of events and details about how the applicant has contributed to science (e.g., articulating exactly what they have done) are also very important.

A question that often arises when preparing a biosketch for a K award has to do with the publication record of the candidate. K award candidates are not expected to have the same or similar a publication record as an R series applicant. However, a publication track record demonstrates that a trainee can take a project through to completion and, as such, is typically valued by reviewers. For the K08/K23 series, some record of original research publication on the topic of the research proposed is often expected. For a K08 applicant, who may be an MD/PhD graduate, evidence of productivity during the PhD phase of training is important and should be emphasized — particularly if the focus of proposed research has not yet yielded a peer-reviewed publication. Additionally, first-author publications, particularly those relevant to the grant topic, should be specifically highlighted. For MD applicants who developed an interest in research during their postgraduate medical training (so called “late bloomers”), a first-author original research publication as a result of postgraduate activity is helpful (though not necessarily always essential); this demonstrates to reviewers not only dedication to research, but also emphasizes the potential for success in a research career. In the current era of peer review, it is important to also note the value of publicly accessible pre-prints that serve not to replace the importance of peer review, but rather to provide reviewers with specific and tangible evidence of productivity that they may or may not elect to evaluate in more detail.

The mentor(s) should also be mindful to write the personal statement in their biosketch specifically for the K award application and not reuse a biosketch they have submitted for an R series or other application. Such an oversight can be viewed as a lack of full engagement of the mentor in the candidate’s research and career development and/or a lack of attention to detail in the preparation of the application. Demonstrating the mentor’s track record of training individuals, especially including K awardees, for successful independent research careers is fundamentally essential to help frame the reviewers’ perceptions of the likely success of the applicant in this mentor-mentee relationship. Experience in leadership and mentoring, such as with training grants (T32, R25, etc.) and inclusion of formal mentorship training, may also be helpful.

### CDP.

The CDP is arguably the most important, yet also often the most frequently marginalized, element of any K award application. Applications that relegate the CDP to little more than a perfunctory set of courses and meetings and retrospective accounting of research skills to be gained often leave the reviewer little recourse other than to evaluate the applicant’s potential to transition to independence based on the likely success of the research plan and record of peer-reviewed publication. Furthermore, CDPs that fail to integrate elements of the mentorship plan and the role of the mentoring team are also often viewed unfavorably. Thus, the CDP should be detailed and must clearly define the need for further training and how the proposed plan will meet these needs and should be closely aligned with other elements of the application (see supplemental materials — K Award Checklist).

Multiple elements can be included, such as coursework, workshops, plans to learn new techniques (especially those that will be essential for the applicant to become independent), networking and presentation opportunities, and intentions to submit future grant applications. Specific (e.g., course numbers, exact dates and times) and realistic details can make the CDP appear more deliberate and concrete to reviewers. A timeline with milestones and accountability is essential, and these are elements that are often less than robust. The CDP is an additional opportunity for the applicant to tell a compelling story about why they decided to pursue a research career (especially if they were not a graduate of an MD-PhD program), and to emphasize the rationale behind the applicant’s selection of the mentoring group and/or an advisory committee that will support the applicant’s career development. Specific details about the role that this group will play in the applicant’s career development are critical, including information about how and how often this group will meet, how it will support the applicant, and how it will hold the applicant and primary mentor(s) accountable for success is of paramount importance. Qualifications and the role that each individual included in the advisory committee will play are likewise important. Details regarding any institutional support and oversight (e.g., through a department or institutional physician-scientist training programs) should also be included.

An important point regarding the applicant’s CDP is that it must match the mentor’s statement. The lack of careful integration of these 2 elements signals to the reviewer that there is either a lack of communication and coordination between the applicant and mentor or that the CDP is not as strong as it could be. It is similarly essential that the CDP be more concretely realistic than pedagogically comprehensive.

### Research plan.

As with all grant proposals, the research plan should be clear and concise, outlining specific aims, background and significance, preliminary data, and the approach. While not expected to be as detailed as an R series research plan, it should be tight and cohesive. Rarely are more than 3 aims necessary. Each aim should ideally serve a specific training goal that can be met independently of the specific scientific outcome associated with that aim. Perhaps most importantly, the applicant and mentor together should ensure that the plan is a good training vehicle and feasible within the proposed timeline and will lead to the applicant developing an independent research career.

The issue surrounding preliminary data is often of interest in CDAs. On one hand, a CDA is intended to help the applicant learn and become adept at generated data, and as such, having large amounts of preliminary data is generally not viewed to be necessary. On the other hand, the presence of preliminary data suggests that the applicant has been active in the laboratory (and likely productive), and many study sections expect to see some amount of preliminary data. Additionally, applicants that generate their own preliminary data are encouraged to point out that they themselves did the work shown.

Elements of the research plan that are often sources of criticism include the failure to address rigor and reproducibility, a complete absence of preliminary data or only preliminary data generated by the laboratory and not the applicant, inadequately addressed statistical design and/or sample size analyses, and a lack of alternative experimental directions.

### Mentor and institutional support.

The primary mentor’s contributions in a K award are paramount. The mentor must have experience in mentoring and ideally should have previous experience in the mentorship of one or more K awardee(s). Of course, every mentor has to start with her/his first K mentee, so in this situation, the evidence that the mentor is qualified to serve as mentor must be incontrovertible. Sometimes co-mentorship with a senior mentor who works closely with the primary mentor may be helpful. Increasingly, mentorship committees are recommended for K awardees. The composition of these committees should be focused on both career development and research direction. Committee members should reflect diversity with respect to institution, scientific interests, and gender, as these individuals will commit time and energy to support the scientific development and progress of the applicant. When co-mentors are used in an application, it must be clear as to which mentor will be fulfilling which role.

It is extremely important to highlight the mentor’s track record in the mentor’s statement and biosketch, particularly if it has been successful. For highly experienced mentors, inclusion of a table listing mentees and what their career outcomes to date have been is useful. Previous mentees with K awards and those who have transitioned to independence should in particular be emphasized.

In order to support a K applicant, the mentor must possess independent extramural funding. Ideally, this funding should have been present for a period of time before the time that the applicant submits the K application — since this signifies an ongoing ability to maintain funding. Additionally, funding should ideally span through at least the start of the proposed K award funding initiation. It is recognized that funding is fluid; nonetheless, the funding history and projected funding of the mentor are important elements of support for the candidate.

Commitment to the career development of the applicant is critical. The mentor letter should detail their own research qualifications, what they will specifically provide, and how they will monitor the applicant’s progress. The mentor should also articulate how they will hold the applicant accountable for meeting milestones proposed in the application. It must be explicitly stated that the applicant’s research program will be independent of the mentors and that the mentor will not compete in this area.

For the advisory committee (or mentoring committee), similar to it being essential that the CDP matches the statement(s) from the mentor(s), it is essential that the letters from these individuals align and serve specific defined goal. For example, if statements about financial, personnel, and space commitment to the applicant, or about scientific aspects of the application are not aligned, reviewers may become concerned about the true degree of commitment of the committee to the applicant’s success.

The institutional environment must support the applicant’s training and research. Letters of support from the institution, in particular, the “chair” letter should emphasize their commitment to the applicant’s success. A strong chair letter is typically a very important aspect of the K award application (see supplemental materials — example of a strong and a weak chair letter, respectively). For most K08 and K23 applications, the chair or institutional letter should make it clear that the commitment to the applicant is not dependent on the funding of the K award. Including a chair letter that promises support that is dependent on K award funding (i.e., in a circular type of argument) is typically viewed very unfavorably by study sections.

### The revised (A1) application – the resubmission.

In the life of a physician-scientist, grant rejection is not uncommon and should not be off-putting to physician-scientists wishing to pursue research careers. NIH K series applications frequently require revisions, as not all applications are funded (Table 4). Before resubmitting the application, the applicant should review the critiques with their mentor and/or mentoring committee to discuss the major strengths and weaknesses highlighted in the reviews and strategize regarding how to address them. After discussing and considering the critiques carefully, the applicant should also contact the program officer to discuss the critiques and planned resubmission.

A response to the reviewer’s comments from the initial (A0) submission should always be included in a 1-page introduction. It is generally important to be as responsive as is possible in this critical aspect of the revised application. Responses should be courteous and professional. It is a good idea to thank the reviewers for their time and constructive critiques and then address each reviewer’s major concerns in a point-by-point manner. Adding a sentence or two at the beginning and end of the introduction including the reviewers’ perceived strengths of the application can remind them that there were a number of positive aspects to the prior submission, not only weaknesses. Many elements are simple to address and correct. Though often helpful, it is no longer allowed to highlight, even in a nonobtrusive manner (e.g., vertical lines beside paragraphs), changes to the application. Therefore changes to the application must be highlighted thoughtfully in the introduction and/or in the application itself. In preparing a revised application, the candidate should strive to ensure that the reviewers can see that the revised application has been responsive to their previous feedback. If one or more reviewer misunderstood an element of the applicant’s proposal, rather than being defensive in the response, it is important to reflect on why this miscommunication occurred. Indeed, it is very easy to address critiques that were simply misunderstandings (10, 11). In some situations, additional data may help provide increased confidence that the proposed set of experiments are the appropriate vehicle for training.

Perhaps what is most important, is to not be discouraged. Reviewers and NIH program officers generally wish to see young physician-scientists given an opportunity to pursue their research aspirations. Rejection is part of every physician-scientist’s career, and resilience is required to overcome it. It is the experience of every successful physician-scientist that persistence almost always pays off.

## Building a sustainable research program from your CDA

Securing a K award is a significant milestone and critical step toward launching an independent research career. Awardees should also use this period to “build their brand” by actively seeking and applying for supplemental funding opportunities compatible with a K award. Pilot grants from institutional centers and programs can provide valuable support and should be aggressively pursued to enhance the research program. Additionally, presenting work at national meetings and accepting invitations to speak at other academic medical centers can increase visibility, foster collaborations, and further establish the awardee’s emerging reputation in the field. Awardees should prioritize generating high-quality, compelling preliminary data to support a competitive application for a major grant, such as an R01, VA MERIT, or similar award. Strategic planning is essential: by the midpoint of the third year of the K award, one should ideally submit both a major grant application and, where eligible, a smaller grant such as an R03 (as permitted by some institutes, including the National Institute of Diabetes and Digestive and Kidney Diseases [NIDDK]). This timing allows for the possibility of resubmission or revision prior to the end of the K award period, facilitating a smoother funding transition. Thoughtful use of the protected time, mentor input, development funds, and research support provided by the K is critical to laying the groundwork for long-term success.

## Miscellaneous tips

### General tips for K award writing.

Reviewers of K applications range in experience and expertise. Some will be highly experienced in reviewing CDAs, others will be recently accomplished (typically an investigator who has just received their first independent extramural research award). Some will have expertise in the subject area of the applicant’s research proposal, and others will not be subject matter experts in the details of every K series research proposal. As such, applicants should anticipate that reviewers of their grant will have general research expertise, but the degree to which they are knowledgeable about the topic of a specific application may vary. Therefore, it is essential to make the application easy to read and to follow. Reviewers all have full-time day jobs, and they may be reviewing at night, on weekends, or while commuting on buses, planes, or trains. Thus, applicants should recognize that there is elegance in simplicity. Some reviewers will read the application on computers or tablets, and some will print. It is usually wise to avoid overly complex experiments, particularly in a K series applications, and it is critically important that the proposal is legible whether reviewed online or printed.

It is strongly recommended to use figures and tables to break up the text. A further tip is that figures, tables, and their legends should be of adequate size to read without difficulty. Only standard abbreviations should be used in a proposal; the last thing a reviewer wants to do when hurried is to spend time trying to understand what an atypical abbreviation means. If uncommon abbreviations are used, it is wise to have an abbreviations box within one of the first pages of the research strategy section that reviewers can rapidly reference. It is also important to eliminate typographical and grammatical errors. If the grant is carelessly written and edited, then a reviewer may infer that the CDP may not be well planned and that scientific oversight may likewise not be rigorous or results reproducible.

Finally, it is always recommended to obtain and to study one or more examples of a successful K series award, preferably from one’s own institution, and preferably one from the applicant’s own mentor. Examples of excellent Biosketches and CDPs are particularly helpful. Many grant writing courses and seminars provide this type of resource.

## Conclusion

Successful grant writing requires clarity, brevity, and a well-articulated plan. It is recommended that a K application demonstrates that the applicant is committed to research, outlines a solid CDP, and is supported by a strong mentoring team and institution. It is also wise for applicants to obtain at least one example of a successful K series award, preferably a similar mechanism, for reference. By following these guidelines, the chances of securing a K series grant will be significantly improved.

## Author contributions

DCR provided study concept and design; drafting of the manuscript; critical revision of the manuscript for important intellectual content; and supervisory efforts. DCR is acting as the submission’s guarantor (i.e., the person who takes responsibility for the integrity of the work as a whole, from inception to published article). KYR provided study concept and design; drafting of the manuscript; and critical revision of the manuscript for important intellectual content. CSW provided study concept and design; drafting of the manuscript; and critical revision of the manuscript for important intellectual content. JMV provided study concept and design; drafting of the manuscript; and critical revision of the manuscript for important intellectual content. CWE provided study concept and design; drafting of the manuscript; and critical revision of the manuscript for important intellectual content. EJG provided study concept and design; drafting of the manuscript; and critical revision of the manuscript for important intellectual content. All authors have approved the final version of the manuscript.

## Supplementary Material

Supplemental data

## Figures and Tables

**Table 1:**
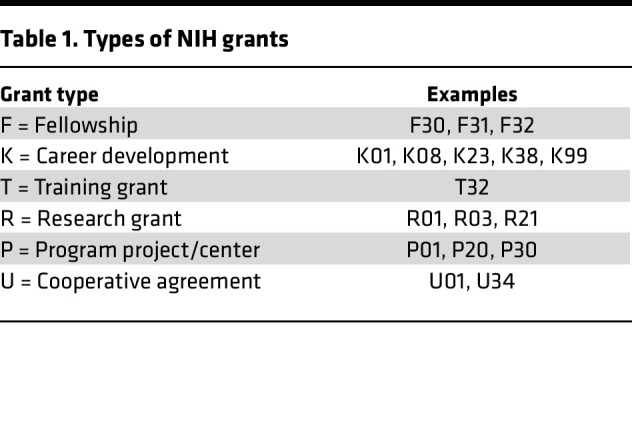
Types of NIH grants

**Table 2:**
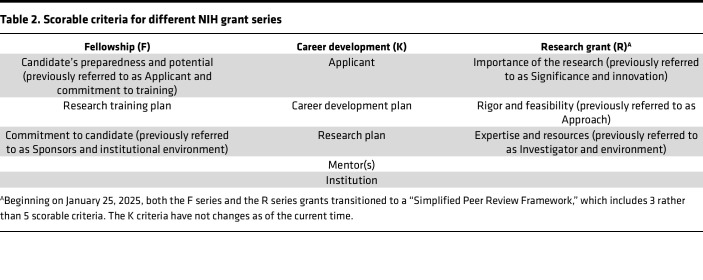
Scorable criteria for different NIH grant series

**Table 3:**
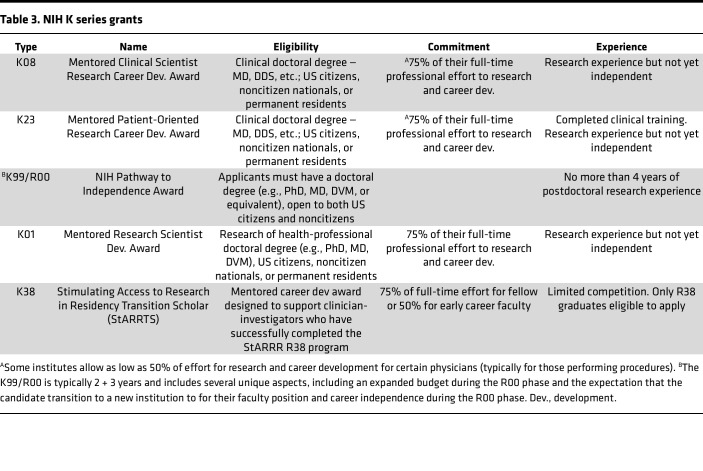
NIH K series grants

**Table 4:**
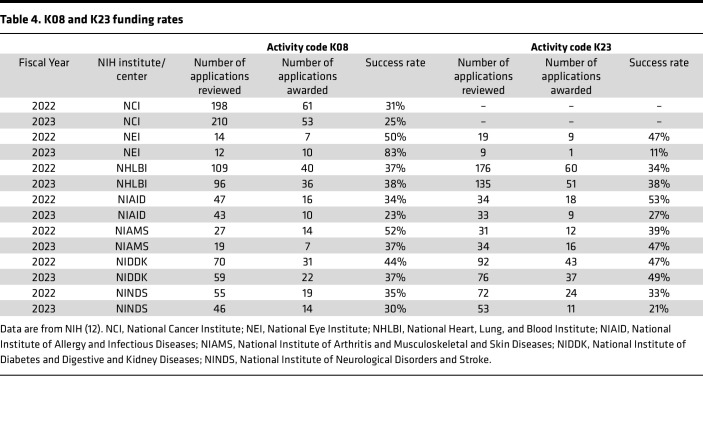
K08 and K23 funding rates

**Table 5:**
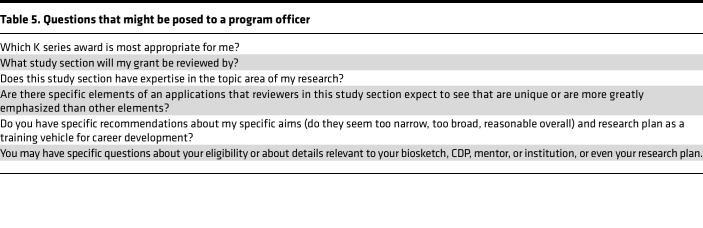
Questions that might be posed to a program officer

## Version 1 06/23/2025

Electronic publication
