# Supplementary material for: An insider’s guide to understanding and obtaining an NIH K career development award
Source: JCI Insight. 2025 Jun 23;10(12):e191904. doi: 10.1172/jci.insight.191904 (PMC12220936; doi:10.1172/jci.insight.191904)
Supplement: Supplemental data [file jciinsight-10-191904-s163.pdf]

Supplemental material for Rockey et al. “An insider’s guide to understanding and obtaining an NIH K career development award”

October 1, 1891

RE: K08 Award for Dr. Adrena Gland

Dear Review Committee:

It is my great pleasure to provide my strongest support to Dr. Adrena Gland, MD, PhD, who is applying for the K08 Career Development Award. This letter documents our institution’s commitment to Adrena and the research and education plan proposed herein. We recruited Adrena to the University of Endocrine where she began her work with us in 1890. She is one of our most beloved new faculty members. Adrena has been a wonderful addition to our faculty and has already been highly successful in her research efforts.

Dr. Gland’s overarching research goal is to become an independent investigator focused on the study of beta-cells in patients with type 1 diabetes mellitus. Our institution is extremely supportive of junior trainees and will certainly support her further career development. Her Division Director, Dr. Thyroid, and I can commit to ensuring that Adrena has the necessary protected time for research. We eagerly anticipate the funding of this K08 award, which will be an essential element of her career development and we undoubtedly plan to promote her to the level of Assistant Professor of Medicine upon its funding. She will also have state of the art laboratory space contained within the space of her primary mentor, Dr. Pituitary.

In summary, we think that Adrena is an outstanding young investigator and has the full support of our institution. We look forward to her career development, and hope that the study section agrees that she is an absolutely outstanding candidate for this K08 career development award.

Signed,

Dr. Chair

Comments:

This weak letter of institutional commitment contains several notable elements:

1. It creates somewhat of a false sense of enthusiasm and commitment for the Applicant. It also uses the first name of the applicant, which is not as professional as using her surname.
2. It does not really provide real support other that protected time, and is very vague about her research space. Reviewers recognize that this generally implies a bench within a PI’s laboratory.
3. This letter clearly lets the reviewers know that the Applicant’s support will be contingent upon obtaining the K08 award, which is not a strong endorsement.
4. The irony with this departmental support letter is that it conveys the message that they, the departmental leadership who know the trainee the best of anyone, will only provide support after a study section, who really does not know the individual at all, deems them worthy of a CDA.
5. The vague statement about protected research time conveys a lack of commitment and doesn’t specifically confirm the NIH-required research time.

Although at face value, this letter is “supportive” and positive about the Applicant - overall, Reviewers will, at best, be luke warm about this letter and Applicant.

Supplemental material for Rockey et al. “An insider’s guide to understanding and obtaining an NIH K career development award”

October 1, 1891

RE: K08 Award for Dr. Adrena Gland

Dear Review Committee:

It is a great pleasure to provide my strongest support to Dr. Adrena Gland, MD, PhD, who is applying for the K08 Career Development Award. This letter documents our institution’s commitment to Dr. Gland and the research and career development plan proposed herein. We recruited Dr. Gland to the University of Endocrine where she began her work in 1890. She is one of our most promising new faculty members. Our commitment to Dr. Gland’s career is not contingent upon this grant, but it is clear that funding of this K08 award will provide her not only with outstanding support, but more importantly, an outstanding training vehicle for her career development as an independent physician-scientist.

Dr. Gland’s overarching research goal is to become an independent investigator with expertise focused on the study of beta-cells in patients with type 1 diabetes mellitus. Our institution brings considerable resources to the table to support her further career development. Her primary mentor is one of the top investigators in the field, who runs an extremely successful laboratory that is known for its welcoming and supportive environment. Additionally, her mentor has guided a number of previous K awardees to independent research careers. In addition, our institution has a number of center grants that will provide the opportunity for her to obtain pilot funding and access to important core facilities.

As Chair, I will ensure that Dr. Gland has 75% of her time protected to perform research. In view of her great potential, we will additionally support her career and its development in the following specific ways:

1. As promised at the time she was promoted to Instructor, when she hit key career benchmarks, which she more than exceeded, she would be promoted to tenure track Assistant Professor, which was done July of 1891, one year after joining the Endocrine Division.
2. She has been assigned dedicated research space (including 500 sq. feet of wet bench space, with shared Departmental space in which to house her -80 Freezer, - 20 Freezer, and an upright refrigerator). Additional space will be provided as she continues the successful expansion of her research program
3. She has also received a start-up package of $750,000 to support her hiring of appropriate personnel and funding of her necessary equipment and supplies.

While we strongly believe that Dr. Gland is an outstanding candidate for a K08 award, our support is not contingent upon its receipt, as we are 100% convinced she “has the right stuff” to continue developing into an world-class independent physician-scientist.

In summary, we believe that Dr. Gland is an outstanding young investigator and has the full support of our institution. We look forward to her career development, and envision her as leader in her field.

Signed, …. Dr. Chair

Comments:

This strong letter of institutional commitment contains several notable elements:

1. It creates a true sense of enthusiasm and commitment for the Candidate.
2. It provides specific and believable deliverables – including needed space, and in particular funding to support establishment of a laboratory.
3. It ensures that the reviewers understand that the Candidate’s support is <u>not</u> contingent upon obtaining the award.

Given these multiple strengths, Reviewers will view this letter as extremely supportive, and score institutional support **very very** well.

Supplemental material for Rockey et al. "An insider's guide to understanding and obtaining an NIH K career development award"

K Award Checklist

This checklist is intended to be relevant for most K awards

Planning

Checklist Element	Suggestions
**Careful review of Notice of Funding Opportunity (NOFO) on NIH websites**	• Ensure that you are applying to the right K award (e.g. K08 for basic science vs K23 for human subjects research).<br>• For resubmission applications, ensure that no new changes to the award or review criteria have been added since the previous submission.
**Allot adequate time for writing of the grant itself**	• Includes ample time for review of grant by your mentor, your advisory committee and others as needed.<br>• A minimum of **6 months of writing and revision time** is suggested, and full grant mentor review is suggested at least 2 months prior to the submission date.
**Chair letter**	• Strategic planning for the chair letter requires discussion with your mentor, your division director and chair. This should occur at least **2 months** prior to the submission date.<br>• See associated supplemental documents for examples of strong and weak chair letters.
**Advisory committee letters**	• You must seek out excellent advisory committee members, and ask them for a letter of support (it is suggested to provide bulleted points for them to incorporate – and these should align with all elements of the training plan.<br>• Letters should be in hand at least **1 month** prior to the submission date.
**Collaborator letters**	• If you have collaborators, obtain letters of support least **1 month** prior to the submission date.<br>• Letters should also be aligned with all elements of the grant.
**Budget**	• The budget for K awards is fixed, but your grants office must have in hand a finished budget as soon as you have decided to write the grant.<br>• The budget should be completed at least **2 months** prior to the submission date.

Grant Submission

Checklist Element	Suggestions
**Candidate**	• Emphasize your experience and why you are the right person for the grant at the right time. Consider the context, for example, the candidate's stage of training and the opportunities available.<br>• Ensure that your and your sponsor's statements as well as the referee letters provide convincing evidence that you possesses qualities (such as scientific understanding, creativity, curiosity, resourcefulness, and drive) that will improve the likelihood of a successful research training outcome.<br>• Emphasize manuscript productivity as appropriate. Having published at least one manuscript in the area of proposed investigation is expected, and depending on your career stage, more may be expected. Having published at least one manuscript with your mentor is often viewed positively.<br>• Reinforce your potential to benefit from the K award and how it will prepare you for a career as an independent investigator.
**Career Development Plan**	• Provide specific details of your plan (see associated supplemental materials that outline key elements), and how the plan will facilitate attainment of your goals.<br>• Focus on areas of needed development and provide appropriate, realistic activities and milestones to address those needs.<br>• Point out how your sponsor(s), scientific environment, facilities, and resources are adequate and appropriate for the proposed career development plan.
**Research Plan**	• Emphasize the rigor and feasibility of the research training project and how completion of the project will contribute your development as an independent investigator.<br>• If you generated preliminary data, emphasize that you generated it yourself.<br>• Discuss whether the research training plan identifies areas of needed development and contains appropriate, realistic activities and milestones to address those needs.<br>• Focus on your sponsor(s), scientific environment, facilities, and resources are adequate and appropriate for the proposed research training plan.
**Mentor(s)**	• The mentoring plan should be appropriate to your needs and goals.<br>• The mentoring plan must align with your career development plan in all details.<br>• Your mentor should have independent extramural funding, and this should be highlighted in the mentors letter and in the mentors biosketch.<br>• Your mentor must provide evidence of effective previous mentoring.<br>• For junior mentors, co-mentors are suggested as appropriate.
**Environment**	• The chair letter must provide evidence of organizational commitment that is appropriate, sufficient, and in alignment with the candidate's

	career development plan.<br>• An appointment to a faculty position is expected at the time the grant is to be funded.<br>• Emphasize how the environment and level of commitment will contribute to the successful completion of your proposed career development and research plan.

During the Award, Post-Award and/or Submission

Checklist Element	Suggestions
**Candidate**	• Publish your work. Having manuscripts published (especially in high quality journals) will greatly support the success rate for an R01.<br>• Consider an R03. For some institutes, a R03 can be submitted during the latter years of the K award. This is intended to provide further funding to prepare for an R01 submission.<br>• Plan an R01 submission. This should occur **during** the K award, preferably beginning in year 2 or 3.
**Resubmission**	• The resubmission application must be responsive to the comments of the Reviewers.<br>• Focus on the major areas of needed improvement and provide appropriate and realistic responses.
**Post Award**	• Begin planning for promotion. Having received a K award (and hopefully an R01 award), you will have had 5 years of experience and should be thinking about being promoted (presumably from Assistant Professor to Associate Professor – at least in most institutions).<br>• See also **Building a Sustainable Research Program from your CDA** in the text

Supplemental material for Rockey et al. “An insider’s guide to understanding and obtaining an NIH K career development award”

Guide to completing a K award **Career Development Plan**

Overview.

The importance of the career development plan of K applications is often underappreciated from not only from the perspective of the application review process but also its contribution to the ultimate success in transitioning to independence. In many cases, this plan is effectively relegated to a retrospective accounting of the specific research activities and experimental methods used in the experiments described in the research plan, supplemented by an either overly broad or narrow didactic curriculum of courses or meetings. However, such plans, while common, often suffer from being limited by the fact that their impact is dependent on the success of the research plan that, unlike the case for R series grants, frequently lies outside the subject matter expertise of the reviewers.

Examination of the K application from the perspective of being a career development award, a key litmus test of all K applications is the likelihood of the proposed training plan to prepare the applicant for the generation of a competitive R01. From this vantage point, such a project-specific training plan is far more risky than one in which the research plan serves to facilitate specific training goals that are independent of the success of the research project itself. As such, it is increasingly the case that K applications with strong career development plans that meet this litmus test are as, if not more, successful than those that 'live or die' on the success of the specific proposed research plan.

CANDIDATE BACKGROUND

Positive Aspects

1. Clearly stated career objective to become an independent physician-scientist / clinician-investigator:
- Defines a specific research area.
- Describes how this research path will have scientific and/or clinical impact by identifying gaps in the field.

2. Cohesive narrative that demonstrates sustained commitment to research:
- Logical progression from early experiences through current work.
- Includes previous mentors and institutions.
- Gaps in research activity or changes in direction are well explained.
- Research is central professional goal.
- Include current position and current protected research time (to demonstrate institution commitment to candidate)

3. Research productivity, and achievements
- Peer-reviewed publications, especially first-author papers as demonstration of ability to complete projects.
- Presentations at regional, and especially national/international meetings.
- Highlights successful prior grants or awards.

4. Articulates training needs:
- Identifies areas where additional training is needed and will be beneficial (e.g., techniques or knowledge) to justify the need for a career development award and mentored support.

Pitfalls to Avoid

1. Disconnect between prior training and proposed research:
- Lack of continuity between past work and future plans.
- Failing to explain any shifts in research direction or gaps in research activity.
- Not mentioning current research activities, and protected time.

2. Not highlighting previous productivity and achievements.
- Not highlighting peer-reviewed publications.
- No mention of previous awards, or grants
- Overreliance on mentors and institutional reputation, without personal achievements
- Vague, non-specific language

3. Inadequate justification for K award:
- Not specifying what skills or training are needed for an independent research career, and how the K award will fill those gaps.

CAREER GOALS AND OBJECTIVES

Positive Aspects

1. Clear, focused career vision:
- Specific long-term goal to become an independent investigator in a defined field.
- Articulates future contributions to research in that field.
- Connects the K award to the future independent research career.

2. Well-defined short-term and long-term goals:
- Short-term: skills acquisition, data generation, publications, training in specific areas.
- Long-term: R01 submission, independent research leadership, mentoring trainees.
- Goals are SMART (Specific, measurable, achievable, relevant and time-bound).

3. Goals and objectives align with mentoring and training plan as well as research strategy:
- Clearly identifies candidate's skill/knowledge gaps.
- Describes clearly how each gap will be addressed.
- Links gaps to training plan, mentors, advisors, planned activities, and institutional resources.

Pitfalls to Avoid

1. Vague long-term career goals:
- Unclear what the candidate will be an expert in.

2. Unattainable goals and/or missing timeline:
- Missing clear short-term and long-term SMART goals and no time-based expectations.

3. No clear training needs
- Not identifying specific skills or knowledge to be acquired.
- Not clearly stating mention of how the K award will fill knowledge or skills gaps.

4. Disconnected from the research plan:
- Goals do not logically align with the proposed project.

5. No mention of R01 or future funding goals:
- The goal of the K award is to prepare the candidate for an independent research career.

CAREER DEVELOPMENT / TRAINING ACTIVITIES

In addition to this career development / training plan, all K award applicants are required to submit a responsible conduct of research training plan. The responsible conduct of research training plan is a separate one page attachment.

Positive Aspects

1. Training plan is specific and aligned with stated goals:
- Training activities are tied directly to skill or knowledge gaps and proposed research aims.
- Training is aligned with progression to independence.
- Training plan is well organized into modules, aligned with research and career goals, tailored to address the stated knowledge or skills gaps.
- Combines mentorship, coursework, seminars, and experiential learning.
- Takes advantage of internal institutional resources and facilities, and external resources as necessary.
- Tables and timelines clearly map training activities to years and deliverables.
- Some K23 grants have specific requirements regarding human subject research, which should included.

2. Strong (complementary) mentorship team:
- Mentors and advisors cover all needed areas of research project, and career goals.
- Clearly defined role of each mentor and advisor.
- Meeting frequency and method (in person, virtual) is clearly stated.
- Some mentors are senior investigators with strong mentoring track records, including mentoring K awardees to independence.

3. Strategic conference participation:
- Conferences selected to support reputation-building and networking.
- Platform for data presentation, research dissemination, feedback, and building collaborations.

4. Includes the development of leadership and mentoring skills:
- Formal training in leadership skills.
- Early introduction to mentoring students and/or fellows.

5. Focus on transition to independence:
- Focused effort to develop a unique, separable research niche.
- Explicit plans to publish independently of mentors.
- Defined timeline for R01 or equivalent submission.
- Intent to own projects/data post-K award.

6. Built-in evaluation process or research and career development progress:
- Regular evaluation meetings to monitor milestone-based progress, and address challenges.
- Creation of an individual development plan (IDP) and mentoring contract (strongly recommended).

7. Clinical integration:
- Clinical responsibilities limited to ≤25% (or ≤50%, if appropriate), with any direct relevance to research clearly stated.

Pitfalls to Avoid

1. Training plan is vague and non-specific:
- Plan does not align with knowledge or skills gaps (or gaps not clearly articulated), and plan does not connect with long term goals, or research strategy.
- Not clear why specific training is needed, how training will be acquired, and no timeline
- Unclear how plan will help candidate transition to independence.

2. K is being used for protected research time only:
- No clear need for K award mentorship or training to fill technical or knowledge gaps.

3. Weak mentorship plan:
- Mentor /advisor roles not clearly defined.
- No clear plan for mentorship meetings.
- Mentor lacking previous experience with K awardees and mentoring in transition to independence.

4. Unfeasible training plan:
- Overly ambitious training plan with too many courses or activities that will detract from completing research project.

5. Missing evaluation plan:
- No individual development plan (IDP).
- No details regarding milestones, and an evaluation plan.

6. No clear path to scientific independence:
- No description of how candidate will develop independent research career.
- No plan for senior authorship.
- No plan or timeline for future independent grant applications.
- No training in mentorship / leadership skills.

7. Missing milestones and/or timeline:
- No description of when training activities, publications and grant submissions will happen.
